# The Effects of Low and High Dose Oral Calcium and Phosphor Supplementation on Nephrocalcinosis Diagnosed by Sonography in Premature and Low Birth Weight Neonates

**Published:** 2014-11

**Authors:** Karmella Kamali, Narjes Pishva, Esmat Deireh

**Affiliations:** 1 Medical Imaging Research Center, Nemazee Hospital, Shiraz University of Medical Science, Shiraz, Shiraz, Iran;; 2 Neonatology Research Center, Neonatal Research Center, Shiraz University of Medical Science, Shiraz, Iran;; 3 Department of Pediatrics, Zeinabieh Hospital, Shiraz University of Medical Sciences, Shiraz, Iran

**Keywords:** Prematurity, Calcium, Phosphor, Nephrocalcinosis, Ultrasonography

## Abstract

Nephrocalcinosis is defined as calcium deposition in the renal interstitium. One of the major causes of neonatal nephrocalcinosis is the use of calcium and phosphor supplements for premature neonates. This study aims at assessing the effects of calcium and phosphor supplementation in neonatal nephrocalcinosis by renal ultrasonography.

In this randomized controlled trial, 37 premature neonates with birth weights <1500 g or a gestational age of <34 weeks were considered. Two different doses of calcium 75 vs. 230 mg/kg/day and phosphor 50 vs. 110 mg/kg/day were prescribed and laboratory and sonographic data were then documented and evaluated.

The incidence of nephrocalcinosis was 47.8% in group 1 and 28.6% in group 2. There was a significant association between NC and positive family history of renal stones, shorter duration of TPN and NICU stay. The amount of calcium dosage, gestational age, birth weight, sex, use of surfactants, and mechanical ventilation did not have any significant association with NC.

In this study, the neonates with NC were mostly the white flake type (8 cases) and the majority of the lesions were 1-2 mm. All the lesions were located in the pyramid and papilla areas, acoustic shadows were not prevalent and stones were not observed in any of the patients.

**Trial Registration Number:** IRCT2013060810441N3

## Introduction

Premature infants undergo intensive growth during the postnatal period. Adequate mineralization depends on sufficient intake of calcium (Ca) and phosphorus (P). However, supplementing Ca and P can be associated with certain risks such as the development of nephrocalconosis.^1^ The term nephrocalcinosis (NC) is used to describe the deposition of calcium in the renal interstitium and is defined as bright reflections in the medulla or cortex seen in both transverse and longitudinal directions.^2^ It may be caused by the imbalance between the promoters and inhibitors of stone formation.^3^^,^^4^ The renal excretory capacity is low due to physiological immaturity of the kidneys. Thus, premature infants are at considerably higher risk of developing nephrocacinosis.^1^ The first case of medullary renal calcification in a premature neonate was reported in 1982.^3^^,^^5^ Since then, NC has become a known complication in neonates, affecting 27 to 65% of premature neonates (weighing under 1500 g) admitted to hospital.^3^

The urine of preterm neonates is generally over-saturated in the first few weeks of life and does not have sufficient ability to control the accumulation of calcium-oxalate. Hypercalciuria is found in 80% of the patients, which indicate its importance as a risk factor for NC.^6^ Consequently, some studies recommend sonographic screening for all neonates receiving diuretics for chronic lung disease or cardiac illness.^3^^,^^4^ The long term effect of this complication remains undetermined in all such studies. However, some researchers have followed up neonates with NC for up to 5-7 years and found no long term association between NC and renal dysfunction.^7^

Currently, many studies aim at assessing the long-term effects of NC on renal function and NC prevention methods in neonates. However, few studies have assessed the effect of calcium and phosphor on neonatal NC. To the best of our knowledge, no similar studies have been done in Iran. Hence, our aim is to assess the incidence of NC following calcium and phosphor supplementation in premature neonates with a gestational age <34 weeks or birth weight <1500 g. It is also intended to investigate the relationship between the doses of calcium therapy and the incidence of NC, and to assess sonographic images of NC. 

## Patients and Methods

**Figure 1:**
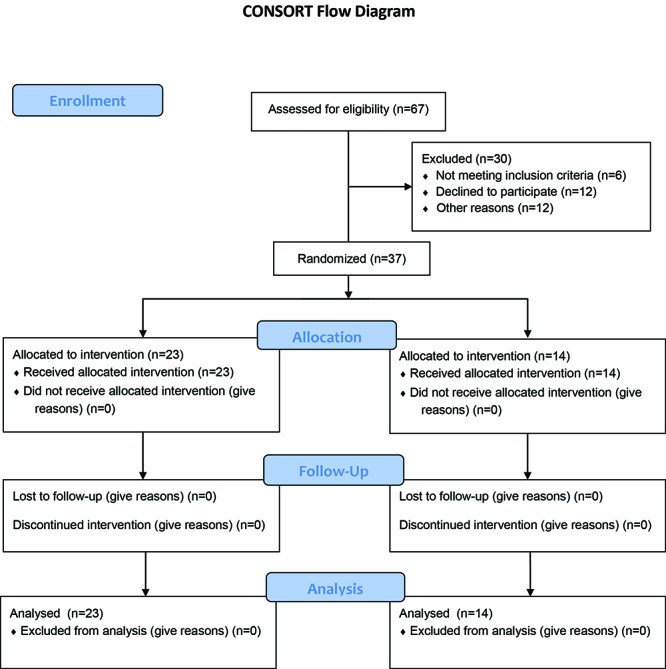
The distribution of patients in two groups.

This randomized controlled trial was done according to a defined timetable on premature neonates being admitted to the intensive care units of Hafez, Nemazee, and Zeinabieh Hospitals during March 2010 to March 2012 (figure 1). These hospitals are affiliated with Shiraz University of Medical Sciences (Shiraz, Iran) and the protocol of this study was approved by the University’s Ethics Committee. The neonates were treated with supplemental calcium and phosphor at two different doses and were evaluated for sonographic signs of NC. Sonography was performed in 30-45, 60, 120, and 360 days after starting the study. If calcification was not present in the previous examination, additional follow up sonography was performed after the above-mentioned sonography plan.

Neonates weighing less than 1500 g or a gestational age under 34 weeks were included in this study. Pending written consent from parents, neonates received 75 and 230 mg/kg doses of calcium and 50, 110 mg/kg/day phosphor during day 10-15 after birth or whenever they could tolerate 10cc breastfeeding. Neonates developing necrotizing enterocolitis after calcium intake, hypercalcemia (serum calcium level of >11 mg/dl) or an increased Cr-BUN level was excluded from the study. If necrotizing enterocolitis and hypercalcemia resolved within 2-3 days, calcium and phosphor supplementation was resumed. 

During the two-year study period, according to a criteria based on the below formula, 67 neonates were included. 

n=([z[α/2]√(2π (1-π))+z[β]√(π_1_(1-π_1_)+π_2_(1-π_2_))]^2^)/δ^2^

Among all the neonates, 37 continued up to the first stage, 17 completed the second stage and 13 managed to complete the third stage of sonography. Ultimately, 37 neonates were randomly enrolled in group 1 (n=23) and group 2 (n=14). The required information and demographic data of these neonates were recorded in the form of a questionnaire. 

Written informed consent was obtained from the neonates’ parents and two different doses of supplemental calcium and phosphor were given to the premature neonates in each group. The first group received 75 and 35 mg/kg/d of supplemental calcium and phosphor, respectively. The second group received 230 and 110 mg/kg/d of supplemental calcium and phosphor, respectively. The amount of calcium and phosphor received through breastfeeding or based on the formula was calculated and subtracted from the amount of supplemental calcium and phosphor. After calcium supplementation, neonates’ serum level of calcium, phosphor, urea, creatinine and electrolyte levels were monitored weekly. Calcium was monitored using the Prestige 24i device (Japan, 2006).

Renal sonography of neonates was performed at intervals of 1, 2, 4 and 12 months after birth. All sonographic evaluations were done by a single expert radiologist at the Department of Radiology at Nemazee Hospital, using General Electric Logic-7 device (USA). The radiologist was kept uninformed on the clinical condition as well as the amount of the calcium and phosphor supplementation of the neonates.

Calcium supplementation was suspended under the following conditions:

1) Serum calcium level more than 11 mg/dl. In such a case, calcium supplementation was stopped for 48-72 hours and if the serum calcium and phosphor amounts returned to normal, the supplementation was restarted.
2) If sonographic findings indicated severe or extensive NC
3) If the neonate reached a weight of 3-3.5 kg
4) If any clinical signs indicating necrotizing enterocolitis such as abdominal distention and vomiting was observed, supplemental calcium was postponed for 48-72 hours.

If calcium deposits were observed in the kidney, the patients would be scheduled for follow-up sonography sessions according to the previously mentioned timetable.

Data were analyzed using SPSS software, version 11. Fisher’s exact test was used to compare the data in both groups. P<0.05 was considered as statistically significant. 

## Results

Among the 37 participants in both groups, 15 (40.5%) neonates developed NC (11 [47.8%] neonates in group 1, and 4 [26.6%] neonates in group 2). There was no significant correlation between the total prevalence of NC and calcium dosage (P=0.314). 

Table 1 shows the relationship between NC and few demographic factors such as weight at birth, gestational age, sex, and family history of renal stones. Out of the seven neonates below the birth weight of 1000 g, two neonates from group 2 developed NC. However, no significant correlation was found between NC and birth weight.

**Table 1:** The relationship between the prevalence of NC and some demographic factors

**Factors**		**Group 1 (n=23)**			**Group 2 (n=14)**			**Total prevalence** **(N, %)**	**Total ** **P value **
		**Developed NC (%)**	**Total**	**P value**	**Developed NC (%)**	**Total**	**P value**		
Birth weight (<1000 g)		0 (0)	3	0.2	2 (50)	4	0.5	2 (26.6)	0.35
Gestational age (weeks) 34 is correct		9 (50)	18	>0.1	4 (28.6)	14	>0.1	13 (40.6)	1
Sex	Girls	6 (46)	13	1	1 (11.1)	9	0.09	7 (31)	0.3
	Boys	5 (50)	10		3 (60)	5		8 (53)	
Family history of renal stones	Negative	6 (33.3)	18	0.014	3 (23)	13	0.2	9 (29)	0.02
	Positive	5 (100)	5		1 (100)	1		6 (100)	

The mean age of the neonates, when oral calcium supplementation was started in those who developed and did not develop NC, were 11.6 and 14.6 days respectively (P=0.13). The mean NICU stay in patients who developed NC was 11.9 days, and 17.7 days for those who did not develop NC (P=0.04). 

In neonates who developed NC, the mean days of Total Parenteral Nutrition (TPN) was 9 days, compared with 16.4 days in the neonates who did not develop NC (P=0.01). Moreover, out of all studied neonates, four neonates received surfactants, and none developed NC (P=0.13). Six neonates underwent mechanical ventilation, and none developed NC (0.06). 

Nineteen neonates did not have acidosis at the beginning of calcium supplementation among which seven developed NC. On the other hand, out of the 18 neonates who had acidosis before calcium supplementation, eight developed NC (P=0.7). 

Laboratory examinations showed that, none of the neonates had increased levels of serum urea and creatinine after calcium and phosphor supplementation. 

Different medications were prescribed for the neonates including furosemide (n=7), theophylline (n=14), dexamethasone (n=7) and vancomycin (n=13). A significant relationship was seen between the prevalence of NC and vancomycin consumption (P=0.04). The consumption of vancomycin significantly reduced the incidence of NC. Moreover, regarding the effect of these medications in both groups, a significant relationship between NC and vancomycin consumption was only seen in group 1 (P=0.014).

Among the neonates in group 1 who underwent the first stage of sonography, 11 neonates did not have NC. Later, one neonate had the symptoms of NC on the second sonography. Eleven neonates had NC on the first sonographic examination, but the signs of NC disappeared in one neonate on the second sonographic examination.

In group 2, 11 neonates had normal finding in the first sonographic examination, only one neonate developed NC according to the second sonographic examination, and three neonates had the signs of NC from the beginning. Sonographic examinations were in average performed at 1-1.5 months after calcium supplementation. The mean age of NC diagnosis in the neonates was 55 days. 

Although the oral doses of calcium and phosphor were high, calcification formation and nephrocalcinosis were minimal and temporary, disappearing during the one year follow up causing no sequela. 

## Discussion

A significant association between NC and positive family history of renal stones, shorter duration of TPN and NICU stay was found. Vancomycin had a significant association with a decrease in the incidence of NC. On the other hand, the amount of calcium dosage, gestational age, birth weight, sex, use of surfactants, mechanical ventilation, and use of furosemide, theophylline, and dexamethasone did not have any significant association with NC.

The reported prevalence of NC in previous studies varies from 16% to 41%.^8^^,^^9^ NC is also observed in 27-65% of premature neonates admitted to hospitals with a birth weight under 1500 g.^3^ Such differences in the prevalence of NC may be due to the discrepancy in the studied populations, ultrasonographic criteria and the sonography device.^10^ In the present study, the prevalence of NC was 40.5% of the total studied population (47.8% in group 1 and 28.6% in group 2). In a recent study on 102 neonates, a relatively small incidence of NC (6%) was reported. Researchers have stated that the low incidence could be associated with possible advances in the neonatal intensive care units and a lesser use of furosemide and corticosteroids (since the 1990s, when most studies reported a high prevalence).^11^

In one study, researchers have concluded that the neonates who developed NC had a lower gestational age and birth weight.^8^ This is inconsistent with another study in which no significant difference was found between NC and birth weight; although a significant relationship still existed between NC and gestational age.^9^ In the present study, the mean weight of the neonates with NC was 1400 g. In general, two out of seven neonates whose birth weights were below 1000 g developed NC (28.6%). Therefore, there was no association between birth weight and NC in both groups.

An insignificant relationship between the prevalence of NC and sex was found which is inconsistent with another related study.^9^ Moreover, in this study, 100% of the neonates with a positive family history of renal stones developed NC. This is contrary to the previously mentioned study, which showed no significant relationship between NC and a positive family history of renal stones.^9^ A forthcoming study from Iran on 49 neonates, reports no significant relationship between the incidence of NC and gestational age, birth weight and sex. In that study, a positive family history of renal stones and high urine calcium/creatinine ratio were significant predictors of NC.^12^

According to the sonographic findings, our study is more consistent with a study performed at the University of Leiden.^13^ Therefore, based on sonographic features; we divided the patients with NC into two groups; patients with white flakes and those with white dots. In the present study, the neonates with NC were mostly the white flake type, and most lesions were 1-2 mm. All the lesions were located in the pyramid and papilla areas, acoustic shadows were not prevalent, and stones were not observed in any of the patients. In most cases, calcium depositions were bilateral and asymmetric, and the lesions were small and benign. The prevalence of NC in the previously mentioned study was 33-41%, and although the dispersion of lesions caused by calcium deposition was high in that study, we found minor lesions. It should also be noted that the lesions related to NC had healed in later serial sonographies, except in one patient who did not refer for the follow-up sonography.

Compared with our study, the patients with NC in a similar study had received TPN for a longer period (18 vs. 7 days for the group that did not develop NC). Another study found no significant relationship between the doses and the volume of TPN and NC.^13^ However, in our study, the patients with NC received TPN for an average of 9 days (versus 16.4 days in patients without NC), and this association was statistically significant.

The effect and the difference of certain factors (e.g. the severity of illness, the wider application of medications in patients with NC, and the difference in TPN solutions used in Iran compared with other countries) could explain why the duration of TPN was less in the present study. It should also be noted that intravenous solutions do not contain calcium. 

Some researchers found a significant association between respiratory diseases, duration of mechanical ventilation, and the use of surfactants with developing NC.^9^ However, no relationship between the duration of the mechanical ventilation and oxygen therapy with NC was reported in other studies.^8^ We did not find a significant relationship between NC and the use of surfactants and mechanical ventilation; although the P value was close to the significant level (P=0.06). This indicates that the relationship might have been significant if a larger sample size was considered.

We did not find a significant relationship between the consumption of furosemide and NC. One study also concluded that there was no significant relationship between the duration of mechanical ventilation, NICU stay, and the duration and frequency of furosemide and steroid consumption between the group with NC and the control group.^14^ However, a recent study showed that furosemide was the strongest independent risk factor for NC with furosemide therapy above 10 mg/kg bodyweight cumulative dose with a 48-fold increased risk of NC. Other predisposing risk factors had an indirect effect.^1^ The risk of renal calcification is higher in low birth weight neonates who are treated with furosemide, dexamethasone, or need long-term ventilation.^11^

The mean age of starting calcium supplementation in our study was 11.6-14.6 days after birth and the mean age at which NC was diagnosed was 55 days. One study performed sonography at six weeks and at term.^8^ There is also a report of performing two sonographic examinations one month after birth and at term or when the neonate was discharged from hospital. On average, we performed sonographic examinations in 1-1.5 months after calcium supplementation.

Although the classification by Saarela^15^ reported the Ca as peripheral, scattered or extensive, calcifications in our study were focused in papillary areas of the medulla, small in size, about 1-2 mm, as flecks or spots, no peripheral or extensive.

## Conclusion

The incidence of nephrocalcinosis using sonography is relatively high, but the depositions are almost benign, scanty and very small. In the present study, the neonates with NC were mostly the white flake type and most lesions were 1-2 mm. Although the oral doses of calcium and phosphor were high, calcification formation and nephrocalcinosis were minimal and temporary disappearing during the one-year follow up causing no sequela. 
